# Depression, Anxiety and Stress Among People Living With HIV: A Cross‑Sectional Study From Antiretroviral Therapy (ART) Centres in Western Rajasthan, India

**DOI:** 10.7759/cureus.102875

**Published:** 2026-02-03

**Authors:** Neha Mantri, Akhil D Goel, Manoj K Gupta, Gopal K Bohra, Nitin K Joshi, Srikant Srinivasan, Siyaram Didel, Rashmi Rathore, Dharamveer Yadav, Naveen Kishoria, Prem Prakash Sharma, Jaykaran Charan, Pankaj Bhardwaj

**Affiliations:** 1 School of Public Health, All India Institute of Medical Sciences, Jodhpur, Jodhpur, IND; 2 Community Medicine & Family Medicine, All India Institute of Medical Sciences, Jodhpur, Jodhpur, IND; 3 Medicine, All India Institute of Medical Sciences, Jodhpur, Jodhpur, IND; 4 Pediatrics, All India Institute of Medical Sciences, Jodhpur, Jodhpur, IND; 5 Dietetics, All India Institute of Medical Sciences, Jodhpur, Jodhpur, IND; 6 Biochemistry, All India Institute of Medical Sciences, Jodhpur, Jodhpur, IND; 7 Medicine, Dr. Sampurnanand Medical College, Jodhpur, IND; 8 Pharmacology, All India Institute of Medical Sciences, Jodhpur, Jodhpur, IND; 9 National Institute for Implementation Research in Non-Communicable Diseases, Indian Council of Medical Research, Jodhpur, IND

**Keywords:** antiretroviral therapies, depression anxiety stress scale, depression screening, hiv aids, india, mental health outcomes, mental health screening, morbidity

## Abstract

Depression, anxiety and stress occur at disproportionately higher rates among people living with HIV (PLWH) compared to the general population. Although antiretroviral therapy (ART) has extended survival, these psychological morbidities remain a critical public health concern. This study aims to estimate the prevalence of depression, anxiety, and stress among PLWH in Western Rajasthan, India, and to identify key sociodemographic and clinical predictors influencing their mental health status.

A cross-sectional study was conducted among 551 PLWH attending two government ART centres between March 2024 and March 2025, using consecutive sampling. Mental health status was assessed through face-to-face administration of the validated Hindi version of the Depression Anxiety and Stress Scale-21 (DASS-21) by trained interviewers. Binary logistic regression was applied to calculate odds ratios (OR) with 95% confidence intervals (CIs), followed by multivariable adjusted analyses.

The prevalence of depression, anxiety, and stress was 11.6%, 19.6%, and 9.6%, respectively. Depression was significantly associated with rural residence (AOR=2.47, 95% CI: 1.02-5.96, p=0.04), absence of spouse (AOR=2.27, 95% CI: 1.03-5.06, p=0.04), and malnutrition (BMI ≤18.5 kg/m²; AOR=3.63, 95% CI: 1.26-10.46, p=0.02). Anxiety was linked to malnutrition (AOR=1.80, p=0.04) and low CD4 count (≤200 cells/mm³; AOR=0.20, p=0.02). Stress was strongly associated with ART nonadherence (≤95%) (AOR=3.77, 95% CI: 1.46-9.72, p<0.01).

These findings underscore the substantial burden of psychological morbidities among PLWH in Western Rajasthan and highlight the need for integrated mental health screening, counselling, and referral mechanisms within HIV care programmes to improve overall health outcomes.

## Introduction

Human immunodeficiency virus (HIV) infection continues to pose a major global public health challenge, with more than 39 million people currently living with HIV (PLWH) worldwide [1]. Beyond its profound immunological consequences, HIV is strongly associated with adverse mental health status. Depression, anxiety, and stress are consistently reported at disproportionately higher prevalence among PLWH compared to the general population, highlighting the need of focus attention. In Ethiopia, for instance, the prevalence of depression among PLWH has been estimated to be nearly twice that observed in the general population [2]. In South Africa, a recent 2025 study reported that 14.5% of people living with HIV experienced depression, compared with 10.8% among HIV-negative individuals [3]. Similarly, studies from the United Kingdom report significantly higher prevalence of depression (17-47% vs. 2-5%), anxiety (22-49% vs. 4-5%), and sleep disturbances (61% vs. 10%) among PLWH compared to the general population [4]. In Eastern Europe and Central Asia, depression prevalence has ranged from 8.5% to 88.0%, while anxiety has varied between 3.0% and 74.0% [5]. Similarly, Indian studies have documented depression rates of 34-61% among PLWH, substantially exceeding estimates of 5-15% in the general population [6,7].

Psychiatric disorders among PLHIV in India are frequently overlooked, with only a minority receiving adequate mental health care. Depression and anxiety are strongly linked to increased morbidity, mortality, poor adherence to antiretroviral therapy (ART), and reduced quality of life [8]. Regional studies have highlighted this burden: Hiremath and Desai reported a 34.5% prevalence of depression among HIV‑positive women in North Karnataka [7], while Savatagi et al. noted that the general health status of PLHIV often reflected distress due to limited healthcare access and interruptions in ART services [9]. Furthermore, Jain et al. demonstrated high prevalence rates of anxiety (59.6%), depression (61.2%), and insomnia (56.4%) among PLHIV in Rohtak, with gender‑specific differences [10]. Moreover, few studies have simultaneously examined both risk factors (e.g., stigma, poor coping, low CD4 counts) and protective factors (e.g., social support, stable ART adherence) influencing mental health status [11,12].

Despite growing evidence, most Indian studies have primarily focused on depression, with limited exploration of anxiety and generalised stress. Given the bidirectional relationship between HIV and mental health [13], untreated psychiatric disorders in PLHIV can accelerate morbidity, contribute to cognitive decline, exacerbate co‑existing health problems, and even result in fatal outcomes such as suicide [14].

Addressing the paucity of data from Rajasthan, particularly Jodhpur, the present study aims to estimate the prevalence of depression, anxiety, and stress using the Depression Anxiety and Stress Scale-21 (DASS‑21) among PLWH attending two-government ART centres and to examine the influence of selected predictors on their mental health status. Findings will provide region‑specific evidence to inform integrated HIV care strategies and strengthen mental health support within ART programmes in Rajasthan.

## Materials and methods

Study design

This was a cross‑sectional study conducted as part of a doctoral research project.

Recruitment and sampling

A consecutive sampling approach was employed to ensure feasibility, minimise non‑response, and capture the experiences of PLWH routinely engaged in care.

Study period

Data were collected between March 2024 and March 2025.

Setting

The study was carried out at two government ART centres in Jodhpur, Western Rajasthan, India. Jodhpur is a major urban city, and the ART centres primarily serve an urban population. However, some participants were drawn from surrounding rural areas who travel to the city for HIV care. Thus, while the study setting was urban and hospital‑based, the participant pool included both urban and rural residents. This mixed urban-rural patient base allowed us to capture diverse sociodemographic and clinical characteristics.

Participants

Eligible participants were HIV‑positive adults aged ≥18 years attending the ART centres during the study period.

Eligibility criteria

Inclusion Criteria

HIV‑positive adults aged ≥18 years who provided written informed consent were included.

Exclusion Criteria

Bed‑bound patients and individuals with severe mental illness who were unable to participate in interviews or complete study tools were excluded.

Sample size

The sample size was calculated using the single proportion formula: n = (Z² * p * (1-p)) / d²

Based on a previous North Indian study reporting a prevalence of poor quality of life among PLHIV as 28.75% [15], the following parameters were applied:

Z=1.96 (for 95% confidence level),

p=0.2875 (prevalence)

(1-p)= 0.7125

d=0.05 (margin of error)

Substituting these values, the minimum required sample size was calculated to be 315. To account for clustering (design effect of 1.5) and an anticipated non‑response rate, the final sample size was determined as 551 participants.

Data collection method

All participants provided written informed consent prior to enrolment, and rapport building was undertaken before data collection commenced. Data were collected through face‑to‑face interviews conducted in Hindi, ensuring cultural and linguistic appropriateness by trained healthcare professionals. Interviews were conducted in private settings within the ART centres, and neutral, non‑judgmental language was used by interviewers to encourage honest reporting. Each interview lasted approximately 20-25 minutes.

Study tools and measures

Data were collected using a pretested and validated structured questionnaire (DASS‑21 for depression, anxiety, and stress), supplemented with sociodemographic and clinical items developed by the investigators. Literacy was assessed by self‑report, wherein participants were asked whether they could read and write in Hindi (the language of the questionnaire and interviews). Those who reported inability to read or write were categorised as illiterate. Socioeconomic status (SES) was assessed using the Modified B.G. Prasad Scale (2024) [16]. Body mass index (BMI) was calculated as weight (kg)/height (m²). WHO clinical staging was applied to classify patients as symptomatic or asymptomatic. CD4+ T-cell counts were recorded to assess immune status. Viral load <200 copies/mL was considered undetectable as per National AIDS Control Organization (NACO) standards.

DASS-21 scale

DASS‑21, a validated 21‑item instrument with three subscales (depression, anxiety, stress), was administered through an interviewer‑read format [17]. The Hindi version of DASS‑21, previously validated for Indian populations [18], was used to ensure linguistic and cultural appropriateness. Participants rated each item on a 4‑point Likert scale reflecting experiences over the past week. Subscale scores were summed and multiplied by “2” to allow comparison with the full 42‑item DASS. The prevalence figures were calculated as the proportion of participants scoring above the “normal” category using the established cut‑off scores:

Depression: 0-9 = normal, 10-13 = mild, 14-20 = moderate, 21-27 = severe, ≥28 = extremely severe

Anxiety: 0-7 = normal, 8-9 = mild, 10-14 = moderate, 15-19 = severe, ≥20 = extremely severe

Stress: 0-14 = normal, 15-18 = mild, 19-25 = moderate, 26-33 = severe, ≥34 = extremely severe

Dependent Variable (Outcome)

The primary outcome was mental health status, assessed as the presence and severity of depression, anxiety, and stress using the validated DASS‑21 scale.

Independent Variables (Predictors)

Sociodemographic factors: Age, sex, marital status, education level, occupation, and socioeconomic status were obtained.

Clinical factors: Duration since HIV diagnosis, ART adherence, CD4 count, viral load, WHO HIV clinical staging, comorbidities, and BMI were recorded.

Sociodemographic and clinical data were routinely collected at ART centres, ensuring reliability, while psychosocial variables were incorporated to capture contextual influences that may uniquely shape mental health in this population. These predictors were selected based on prior evidence linking them to mental health outcomes among PLWH and their contextual relevance in Western Rajasthan.

Statistical analysis

Data were collected using Epicollect 5 software and imported into IBM SPSS Statistics version 23 (SPSS Inc., Armonk, NY, USA) for analysis. Regarding data quality assurance, the questionnaire was pretested to refine wording and reduce ambiguity, and interviewers received training and supervision to ensure uniform administration. Completed forms were reviewed daily for accuracy, and double data entry with consistency checks was performed to minimise errors. For analysis, missing data were handled using listwise deletion when <5% of responses were missing.

Descriptive statistics were presented as frequencies, percentages, means (standard deviations), and medians (interquartile ranges). Associations between categorical predictors and DASS categories were examined using simple logistic regression. Model fit was evaluated using the Hosmer-Lemeshow goodness‑of‑fit test for logistic regression, which showed acceptable fit. Odds ratios (ORs) with 95% confidence intervals (CIs) were reported, and statistical significance was defined as p < 0.05. Variables with p < 0.20 in univariate analysis were included in the multivariable model [19].

Ethical considerations

Ethical approval was obtained from the Institutional Ethics Committee of All India Institute of Medical Sciences, Jodhpur (Ref. No. AIIMS/IEC/2023/5984). Written informed consent was obtained from all participants before enrollment, following a detailed explanation of the study objectives, procedures, and confidentiality safeguards. For participants with limited literacy, the consent form was read aloud in their preferred language, and consent was documented with a thumb impression in the presence of a witness. Participants who presented with psychosocial comorbidities received counselling interventions and were duly referred to appropriate support services to ensure continuity of care.

## Results

A total of 551 PLWH were enrolled in the study, comprising 320 male participants (58.1%) and 231 female participants (41.9%) with a mean age of 42.9 ± 12.4 years. Among the participants, 381 (69.1%) were married, 469 (85.1%) resided in urban areas, and 350 (63.5%) lived in nuclear families. With respect to educational status, 240 participants (43.6%) were illiterate. Socio-economic classification showed that 347 (63.0%) belonged to the lower socio-economic class. The predominant route of HIV transmission was heterosexual contact, reported in 261 participants (47.3%). Clinical staging revealed that 461 participants (83.7%) were asymptomatic and classified under WHO stage I. Comorbidities were present in 112 individuals (20.3%). The median CD4 count was 467 cells/mm³ (IQR: 265-678), and viral load was undetectable in 478 participants (86.8%). The detailed characteristics of the study participants are presented in Table 1.

**Table 1:** Gender-wise distribution of HIV-related variables among PLWH (N=551) PLWH: People Living with HIV

Variables	Female, n (%)	Male, n (%)
Age category (years)		
15–29	38 (44.7)	47 (55.3)
30–44	98 (45.0)	120 (55.0)
45–60	73 (39.7)	111 (60.3)
≥ 60	22 (34.4)	42 (65.6)
Typology		
General	217 (44.0)	276 (56.0)
Pregnant	6 (100.0)	0 (0.0)
Injecting drug user (IDU)	0 (0.0)	3 (100.0)
Long-distance truckers	0 (0.0)	32 (100.0)
Migrant	6 (37.5)	10 (62.5)
Preferred not to answer	1 (100.0)	0 (0.0)
Marital status		
Unmarried	6 (11.1)	48 (88.9)
Married	134 (35.2)	247 (64.8)
Divorced/Separated	2 (40.0)	3 (60.0)
Widowed	88 (80.0)	22 (20.0)
Preferred not to answer	1 (100.0)	0 (0.0)
Education		
No formal education	152 (63.3)	88 (36.7)
Formal education	79 (25.4)	232 (74.6)
Occupation		
Unskilled/Semi-skilled	213 (56.2)	166 (43.8)
Skilled	18 (10.4)	154 (89.6)
Socio-economic status		
Class I (Upper)	0 (0.0)	2 (100.0)
Class II (Upper Middle)	7 (9.7)	65 (90.3)
Class III (Middle)	23 (17.7)	107 (82.3)
Class IV (Lower Middle)	104 (43.3)	136 (56.7)
Class V (Lower)	97 (90.7)	10 (9.3)
Residence		
Rural	35 (42.7)	47 (57.3)
Urban	196 (41.8)	273 (58.2)
Type of family		
Nuclear	136 (38.9)	214 (61.1)
Joint	95 (47.3)	106 (52.7)
WHO clinical staging		
Asymptomatic	207 (44.9)	254 (55.1)
Symptomatic	24 (27.0)	65 (73.0)
AIDS converted	0 (0.0)	1 (100.0)
Time since diagnosis		
< 1 year	74 (37.9)	121 (62.1)
1–5 years	81 (42.4)	110 (57.6)
> 5 years	76 (46.1)	89 (53.9)
Drug adherence		
Non-adherent	41 (45.1)	50 (54.9)
Adherent	190 (41.3)	270 (58.7)
Body mass index (BMI)		
< 18.5 kg/m²	49 (47.1)	55 (52.9)
18.5–22.9 kg/m²	120 (45.3)	145 (54.7)
≥ 23.0 kg/m²	62 (34.1)	120 (65.9)
CD4 count		
< 200 cells/mm³	37 (40.2)	55 (59.8)
≥ 200 cells/mm³	181 (43.9)	231 (56.1)
Viral load		
> 200 copies/ml	14 (58.3)	10 (41.7)
≤ 200 copies/ml	200 (41.8)	278 (58.2)

Mental health status

The DASS‑21 demonstrated excellent internal consistency overall (Cronbach’s alpha = 0.911). Subscale reliabilities were acceptable: Depression (α = 0.778), Anxiety (α = 0.780), and Stress (α = 0.763), indicating that each domain was measured consistently within the study sample. Mild symptoms were reported by 30 (5.4%) participants for depression, 36 (6.5%) for anxiety, and 34 (6.2%) for stress. Moderate levels were observed in 24 (4.4%) for depression, 38 (6.9%) for anxiety, and 15 (2.7%) for stress. Severe symptoms were present in 7 (1.3%) for depression, 16 (2.9%) for anxiety, and 4 (0.7%) for stress. Finally, extreme severity was noted in two (0.4%) participants for depression and 18 (3.3%) for anxiety, while none reported extreme stress. The mental health status is described in Table 2.

**Table 2:** Distribution of depression, anxiety, and stress levels among PLWH (N=551) Values are presented as number of participants (N) with corresponding percentages (%). PLWH: People living with HIV

Level	N (%)
Depression	
Normal	488 (88.6)
Mild	30 (5.4)
Moderate	24 (4.4)
Severe	7 (1.3)
Extreme severe	2 (0.4)
Anxiety	
Normal	443 (80.4)
Mild	36 (6.5)
Moderate	38 (6.9)
Severe	16 (2.9)
Extreme severe	18 (3.3)
Stress	
Normal	498 (90.4)
Mild	34 (6.2)
Moderate	15 (2.7)
Severe	4 (0.7)
Extreme severe	0 (0.0)

Association of depression and its predictors among PLWH

In the univariate analysis, rural residence, absence of a spouse, and malnutrition (low BMI) emerged as significant correlates of depression among PLWH. Other socio-demographic, clinical, and treatment-related variables did not show statistically significant associations.

On multivariable logistic regression, depression was significantly associated with rural residence (AOR: 2.47, 95% CI: 1.02-5.96, p=0.04), absence of formal education (AOR: 0.40, 95% CI: 0.17-0.93, p=0.03), spouse absence (AOR: 2.27, 95% CI: 1.03-5.06, p=0.04), and malnutrition (BMI ≤18.5 kg/m²) (AOR: 3.63, 95% CI: 1.26-10.46, p=0.02). The findings are presented in Table 3.

**Table 3:** Association of depression and its predictors among PLWH (n=551) Values are presented as number of participants (n) with corresponding percentages (%). OR: Odds Ratio; CI: Confidence Interval; Ref: Reference category; PLWH: People Living with HIV. Unadjusted ORs were derived from bivariate logistic regression, while adjusted ORs were obtained from multivariable logistic regression including variables significant at p < 0.05

Variable	Category	Depression Present, n (%)	Depression Absent, n (%)	Unadjusted OR (95% CI)	Adjusted OR (95% CI)
Age (years)	>30	28 (6.2)	424 (93.8)	1.24 (0.46–3.35)	–
	≤30 (Ref)	5 (5.1)	94 (94.9)	Ref	Ref
Sex	Male	21 (6.6)	299 (93.4)	1.29 (0.63–2.63)	–
	Female (Ref)	12 (5.2)	219 (94.8)	Ref	Ref
Residence	Rural	9 (11.0)	73 (89.0)	2.29 (1.03–5.09)	2.47 (1.02–5.96)
	Urban (Ref)	24 (5.1)	445 (94.9)	Ref	Ref
Education	No formal education	11 (4.6)	229 (95.4)	1.60 (0.76–3.37)	0.40 (0.17–0.93)
	Formal education (Ref)	22 (7.1)	289 (92.9)	Ref	Ref
Occupation	Unskilled/Semi-skilled	23 (6.1)	356 (93.9)	1.06 (0.49–2.29)	–
	Skilled (Ref)	10 (5.8)	162 (94.2)	Ref	Ref
Socioeconomic status	Class IV–V	22 (6.3)	325 (93.7)	1.18 (0.55–2.53)	–
	Class I–III (Ref)	11 (5.4)	193 (94.6)	Ref	Ref
Marital status	Spouse absent	16 (9.4)	154 (90.6)	2.21 (1.07–4.56)	2.27 (1.03–5.06)
	Spouse present (Ref)	17 (4.5)	364 (95.5)	Ref	Ref
Family type	Nuclear	20 (5.7)	330 (94.3)	1.15 (0.56–2.37)	–
	Joint (Ref)	13 (6.5)	188 (93.5)	Ref	Ref
BMI classification	Malnourished (≤18.5 kg/m²)	29 (7.9)	340 (92.1)	3.80 (1.32–10.97)	3.63 (1.26–10.46)
	Normal (>18.5 kg/m²) (Ref)	4 (2.2)	178 (97.8)	Ref	Ref
Comorbidity	Present	8 (7.1)	104 (92.9)	1.27 (0.56–2.87)	–
	Absent (Ref)	25 (5.7)	414 (94.3)	Ref	Ref
WHO clinical stage	Symptomatic (II–IV)	5 (5.6)	85 (94.4)	1.10 (0.41–2.94)	–
	Asymptomatic (I) (Ref)	28 (6.1)	433 (93.9)	Ref	Ref
Adherence	Non-adherent (≤95%)	7 (7.7)	84 (92.3)	1.39 (0.59–3.26)	–
	Adherent (>95%) (Ref)	26 (5.7)	434 (94.3)	Ref	Ref
ART duration	>5 years	9 (5.5)	156 (94.6)	0.88 (0.40–1.94)	–
	≤5 years (Ref)	24 (6.2)	362 (93.8)	Ref	Ref
CD4 count (cells/mm³)	≤200	3 (3.3)	89 (96.7)	0.51 (0.15–1.74)	0.46 (0.13–1.59)
	>200 (Ref)	26 (6.3)	386 (93.7)	Ref	Ref
Viral load	>200 copies/mL	2 (8.3)	22 (91.7)	1.40 (0.31–6.32)	–
	≤200 copies/mL (Ref)	29 (6.1)	449 (93.9)	Ref	Ref

Association of anxiety and its predictors among PLWH

In the univariate analysis, marital status (absence of spouse), BMI classification (malnutrition), and CD4 cell count (≤200 cells/mm³) were significantly associated with anxiety among PLWH. On multivariable logistic regression, anxiety was significantly associated with marital status (absence of spouse), BMI classification and CD4 cell count. PLWH without a spouse had higher odds of experiencing stress compared to those with a spouse (AOR = 2.27, 95% CI: 1.03-5.06, p = 0.04). PLWH who were malnourished (BMI ≤18.5 kg/m²) had higher odds of anxiety (AOR: 1.80, 95% CI: 1.00-3.54, p=0.04). Similarly, those with CD4 counts ≤200 cells/mm³ were at increased risk (AOR: 0.20, 95% CI: 0.10-0.82, p=0.02), indicating a protective effect of higher CD4 counts. The findings are presented in Table 4.

**Table 4:** Association of anxiety and its predictors among PLWH (n=551) Values are presented as number of participants (n) with corresponding percentages (%). OR: Odds Ratio; CI: Confidence Interval; Ref: Reference Category; PLWH: People Living with HIV. Unadjusted ORs were derived from bivariate logistic regression, while adjusted ORs were obtained from multivariable logistic regression including variables significant at p < 0.05

Variable	Category	Stress Present, n (%)	Stress Absent, n (%)	Unadjusted OR (95% CI)	Adjusted OR (95% CI)
Age (years)	>30	61 (13.5)	391 (86.5)	1.25 (0.62–2.53)	–
	≤30 (Ref)	11 (11.1)	88 (88.9)	Ref	Ref
Sex	Male	41 (12.8)	279 (87.2)	0.95 (0.56–1.61)	–
	Female (Ref)	31 (13.4)	200 (86.6)	Ref	Ref
Residence	Rural	12 (14.6)	70 (85.4)	0.86 (0.44–1.67)	–
	Urban (Ref)	60 (12.8)	409 (87.2)	Ref	Ref
Education	No formal education	32 (13.3)	208 (86.7)	0.97 (0.58–1.62)	–
	Formal education (Ref)	40 (12.9)	271 (87.1)	Ref	Ref
Occupation	Unskilled/Semi-skilled	50 (13.2)	329 (86.8)	0.97 (0.56–1.67)	–
	Skilled (Ref)	22 (12.8)	150 (87.2)	Ref	Ref
Socioeconomic status	Class IV–V	42 (12.1)	305 (87.9)	1.26 (0.77–2.05)	–
	Class I–III (Ref)	30 (14.7)	174 (85.3)	Ref	Ref
Marital status	Spouse absent	30 (17.6)	140 (82.4)	1.74 (1.04–2.91)	2.27 (1.03–5.06)
	Spouse present (Ref)	42 (11.0)	339 (89.0)	Ref	Ref
Family type	Nuclear	41 (11.7)	309 (88.3)	1.39 (0.84–2.29)	0.80 (0.47–1.41)
	Joint (Ref)	31 (15.4)	170 (84.6)	Ref	Ref
BMI classification	≤18.5 kg/m²	56 (15.2)	313 (84.8)	1.87 (1.05–3.34)	1.80 (1.00–3.54)
	>18.5 kg/m² (Ref)	16 (8.8)	166 (91.2)	Ref	Ref
Comorbidity	Present	15 (13.4)	97 (86.6)	1.04 (0.56–1.93)	–
	Absent (Ref)	57 (13.0)	382 (87.0)	Ref	Ref
WHO clinical stage	Symptomatic (II–IV)	12 (13.3)	78 (86.7)	1.03 (0.52–2.03)	–
	Asymptomatic (I) (Ref)	60 (13.0)	401 (87.0)	Ref	Ref
Adherence	Non-adherent (≤95%)	16 (17.6)	75 (82.4)	1.54 (0.83–2.84)	1.40 (0.76–2.86)
	Adherent (>95%) (Ref)	56 (12.2)	404 (87.8)	Ref	Ref
ART duration	>5 years	28 (17.0)	137 (83.0)	1.59 (0.95–2.66)	1.50 (0.90–2.73)
	≤5 years (Ref)	44 (11.4)	342 (88.6)	Ref	Ref
CD4 count (cells/mm³)	≤200	4 (4.3)	88 (95.7)	0.27 (0.09–0.78)	0.20 (0.10–0.82)
	>200 (Ref)	59 (14.3)	353 (85.7)	Ref	Ref
Viral load	>200 copies/mL	3 (12.5)	21 (87.5)	0.92 (0.26–3.23)	–
	≤200 copies/mL (Ref)	64 (13.4)	414 (86.6)	Ref	Ref

Association of stress and its predictors among PLWH

On multivariable logistic regression, nonadherence to ART (≤95%) was significantly associated with stress (AOR: 3.77, 95% CI: 1.46-9.72, p<0.01). Participants who were non-adherent had 3.7 times higher odds of reporting stress compared to adherent individuals. The findings are presented in Table 5.

**Table 5:** Association of stress and its predictors among PLWH (n=551) Values are presented as number of participants (n) with corresponding percentages (%). OR: Odds Ratio; CI: Confidence Interval; Ref: Reference Category; PLWH: People Living with HIV. Unadjusted ORs were derived from bivariate logistic regression, while adjusted ORs were obtained from multivariable logistic regression including variables significant at p < 0.05

Variable	Category	Stress Present, n (%)	Stress Absent, n (%)	Unadjusted OR (95% CI)	Adjusted OR (95% CI)
Age (years)	>30	16 (3.5)	436 (96.5)	1.18 (0.33–4.15)	–
	≤30 (Ref)	3 (3.0)	96 (97.0)	Ref	Ref
Sex	Male	12 (3.8)	308 (96.3)	1.28 (0.49–3.34)	–
	Female (Ref)	7 (3.0)	224 (97.0)	Ref	Ref
Residence	Rural	4 (4.9)	233 (97.1)	1.57 (0.49–4.99)	–
	Urban (Ref)	15 (3.2)	299 (96.1)	Ref	Ref
Education	No formal education	7 (2.9)	233 (97.1)	1.34 (0.52–3.36)	–
	Formal education (Ref)	12 (3.9)	299 (96.1)	Ref	Ref
Occupation	Unskilled/Semi-skilled	14 (3.7)	365 (96.3)	1.29 (0.47–3.55)	–
	Skilled (Ref)	5 (2.9)	167 (97.1)	Ref	Ref
Socioeconomic status	Class IV–V	13 (3.7)	334 (96.3)	1.29 (0.49–3.39)	–
	Class I–III (Ref)	6 (2.9)	198 (97.1)	Ref	Ref
Marital status	Spouse absent	8 (4.7)	162 (95.3)	1.67 (0.65–4.28)	–
	Spouse present (Ref)	11 (2.9)	370 (97.1)	Ref	Ref
Family type	Nuclear	13 (3.7)	337 (96.3)	1.25 (0.48–3.28)	–
	Joint (Ref)	6 (3.0)	195 (97.0)	Ref	Ref
BMI classification	≤18.5 kg/m²	15 (4.1)	354 (95.9)	1.91 (0.62–5.89)	1.84 (0.59–5.66)
	>18.5 kg/m² (Ref)	4 (2.2)	178 (97.8)	Ref	Ref
Comorbidity	Present	4 (3.6)	108 (96.4)	1.05 (0.34–3.23)	–
	Absent (Ref)	15 (3.4)	424 (96.6)	Ref	Ref
WHO clinical stage	Symptomatic (II–IV)	5 (5.6)	85 (94.4)	1.92 (0.68–5.43)	1.60 (0.54–4.63)
	Asymptomatic (I) (Ref)	14 (3.0)	447 (97.0)	Ref	Ref
Adherence	Non-adherent (≤95%)	8 (8.8)	83 (91.2)	3.95 (1.52–10.20)	3.77 (1.46–9.72)
	Adherent (>95%) (Ref)	11 (2.4)	449 (97.6)	Ref	Ref
ART duration	>5 years	6 (3.6)	159 (96.4)	1.06 (0.40–2.82)	–
	≤5 years (Ref)	13 (3.4)	373 (96.6)	Ref	Ref
CD4 count (cells/mm³)	≤200	0 (0.0)	92 (100.0)	–	–
	>200 (Ref)	16 (3.9)	396 (96.1)	Ref	Ref
Viral load	>200 copies/mL	1 (4.2)	23 (95.8)	1.91 (0.62–5.89)	–
	≤200 copies/mL (Ref)	17 (3.6)	461 (96.4)	Ref	Ref

## Discussion

Depression, stress, and anxiety represent psychological morbidities that significantly compromise occupational functioning, social relationships, and overall quality of life. Evidence suggests that their impact may exceed that observed in individuals with other chronic illnesses such as congestive heart failure, diabetes, or myocardial infarction [20]. Within the context of PLWH, these mental health concerns assume particular importance, as antiretroviral therapy has successfully extended survival yet has not alleviated the persistent burden of psychological distress. The present study contributes to this body of evidence by situating its findings within both global and Indian contexts, highlighting the prevalence and key predictors of depression, stress, and anxiety among PLWH that pose a significant barrier to optimal health outcomes.

Depression: prevalence and predictors

In this study, 11.4% of PLWH reported depression, a prevalence notably lower than that observed in a recent systematic review, which estimated a pooled prevalence of 39.1% depression in India [21]. Univariate analysis further revealed that male PLWH were at greater risk of depression, aligning with findings from Peru among middle‑aged individuals living with HIV [22]. Globally, however, depression is more common among women, with nearly double the odds (OR: 1.95) compared to men [23]. These differences may reflect gender‑related stigma and social isolation faced by men in the Indian context, which can exacerbate psychological distress. Residence was a significant determinant of depression. PLWH in rural areas had higher odds of depression compared to urban residents (OR: 2.29), which may stem from limited healthcare access, greater stigma, or underreporting biases, consistent with evidence from sub-Saharan Africa [24].

Although univariate analysis showed no significant association, multivariate analysis revealed that formal education reduced the odds of depression (AOR: 0.40). Hence, formal education was related to lower odds of depression by improving health literacy, coping strategies, and engagement with HIV care, thereby lowering vulnerability to depression [25]. PLWH without a spouse had 2.2 times higher odds of depression compared to those with a spouse (OR: 2.21). The absence of spousal support may reduce emotional stability and coping capacity. Studies from Indonesia highlighted that marital support enhances quality of life and reduces depressive symptoms among PLWH [26].

In our study, malnutrition was associated with higher odds of depression. Malnourished participants (BMI ≤18.5 kg/m²) had higher odds of depression compared to those with normal BMI (AOR: 3.63). This may be attributed to nutritional deficiencies, impaired immunity, and the bidirectional cycle between poor nutrition and depression. Evidence from African countries confirms that undernutrition is highly prevalent among PLWH and is strongly associated with depression and poor ART adherence [27].

Anxiety: prevalence and predictors

The prevalence of anxiety among PLWH in our cohort was 19.6%, similar to rates reported in other low‑ and middle‑income settings [28]. Nutritional status emerged as a significant predictor: malnourished participants (BMI ≤18.5 kg/m²) had nearly twice the odds of anxiety compared to those with normal BMI (AOR: 1.80, 95% CI: 1.00-3.54, p = 0.04). Similarly, CD4 cell count was strongly associated with anxiety. Participants with CD4 ≤200 cells/mm³ had markedly lower odds of anxiety compared to those with higher counts (AOR: 0.20, 95% CI: 0.10-0.82, p = 0.02). This relationship may reflect that PLWH with advanced immunosuppression often experience reduced affective reactivity or prioritise physical illness over psychological symptoms, as reported in prior studies [29].

Stress: prevalence and predictors

In this study, the prevalence of stress was 9.7% among PLWH. The non‑adherent PLWH (≤95% adherence) had higher odds of experiencing stress compared to those who were adherent (>95%) (AOR: 3.77). Poor adherence may contribute to increased viral replication, immune suppression, and fear of disease progression, all of which heighten psychological distress. These findings are consistent with prior studies from a systematic review, which reported that non‑adherence to ART is strongly linked to higher levels of stress, anxiety, and depression among PLWH [30]. However, residual confounding by psychosocial factors such as stigma and coping strategies cannot be ruled out. Conversely, stress itself can negatively impact adherence by impairing concentration, motivation, and routine medication intake, creating a bidirectional cycle between stress and adherence.

Strength and limitations

A key strength of this study lies in its focus on the mental health of PLWH in India, an area often underexplored despite its critical importance. The use of standardised measures to assess depression, anxiety, and stress, along with the inclusion of socio-demographic, nutritional, and immunological factors, provides a comprehensive understanding of determinants influencing psychological morbidity.

Several limitations of this study must be acknowledged. First, the cross-sectional design limits causal inference, and residual confounding by unmeasured psychosocial factors (e.g., stigma, coping mechanisms, social support) may partly explain these associations. Second, reliance on self‑reported measures may have introduced recall and social desirability bias, despite efforts to minimise these through private interviews and neutral language. Third, external validity was limited to PLWH attending government ART centres in Jodhpur, and findings may not be generalizable to private facilities or community‑based settings; future multi‑centric research across diverse contexts would enhance generalizability. Fourth, temporal ambiguity remains, as it is unclear whether depression preceded or followed the identified risk factors. Fifth, variation in definitions and classification criteria for depression across studies may have led to misclassification. Sixth, although missing data were minimised, the inability to perform sensitivity analysis is noted as a methodological limitation, potentially affecting the robustness of findings. Regarding multiple comparisons, we have noted that analyses were exploratory in nature and no formal adjustment was applied. Finally, while quantitative measures provided prevalence estimates and predictors, incorporating qualitative methods such as focus group discussions or in‑depth interviews could yield deeper insights into the psychosocial dimensions of living with HIV.

Policy implications and future directions

This study underscores the need to integrate routine mental health screening for depression, anxiety, and stress into HIV care, with targeted support for vulnerable subgroups. Regular monitoring of BMI and CD4 counts, alongside nutritional, psychosocial, and adherence counselling interventions, can mitigate psychological morbidity and improve quality of life for PLWH.

Future research should explore longitudinal associations of ART adherence, treatment duration, BMI, and comorbidities with mental health outcomes. We hypothesise that consistent ART adherence and longer treatment duration will lower the incidence and severity of depression, anxiety, and stress, while poor adherence, comorbidities, or malnutrition will heighten risk.

## Conclusions

In this cohort of PLWH, the prevalence of depression, anxiety, and stress was lower than some previously reported estimates but remained consistently higher than levels observed in the general population, underscoring the persistent burden of psychosocial morbidity in this group. Mental health outcomes were influenced by selected sociodemographic and clinical factors, with rural residence, spousal absence, malnutrition, underweight status, and low CD4 counts emerging as important correlates. Stress was most strongly associated with non-adherence to ART, highlighting the bidirectional relationship between psychological well-being and treatment outcomes.

Although subgroup differences were modest, the findings emphasise that vulnerability to mental health problems among PLWH is multifactorial and not confined to specific demographic or clinical categories. The null associations with age, sex, socioeconomic status, comorbidities, and WHO staging suggest that broader contextual and structural influences may play a critical role. These results reinforce the need for integrated approaches that combine routine mental health screening with targeted interventions addressing nutritional status, social support, and ART adherence, thereby strengthening holistic HIV care and improving long-term outcomes.
